# Morpholino-Mediated Increase in Soluble Flt-1 Expression Results in Decreased Ocular and Tumor Neovascularization

**DOI:** 10.1371/journal.pone.0033576

**Published:** 2012-03-15

**Authors:** Leah A. Owen, Hironori Uehara, Judd Cahoon, Wei Huang, Jacquelyn Simonis, Balamurali K. Ambati

**Affiliations:** Department Of Ophthalmology, Moran Eye Center, University of Utah, Salt Lake City, Utah, United States of America; University of Kentucky, United States of America

## Abstract

**Background:**

Angiogenesis is a key process in several ocular disorders and cancers. Soluble Flt-1 is an alternatively spliced form of the *Flt-1* gene that retains the ligand-binding domain, but lacks the membrane-spanning and intracellular kinase domains of the full-length membrane bound Flt-1 (mbFlt-1) protein. Thus, sFlt-1 is an endogenous inhibitor of VEGF-A mediated angiogenesis. Synthetic mopholino oligomers directed against splice site targets can modulate splice variant expression. We hypothesize that morpholino-induced upregulation of sFlt-1 will suppress angiogenesis in clinically relevant models of macular degeneration and breast cancer.

**Methods and Findings:**

*In vivo* morpholino constructs were designed to target murine exon/intron 13 junction of the Flt-1 transcript denoted VEGFR1_MOe13; standard nonspecific morpholino was used as control. After nucleofection of endothelial and breast adenocarcinoma cell lines, total RNA was extracted and real-time RT-PCR performed for sFlt-1 and mbFlt-1. Intravitreal injections of VEGFR1_MOe13 or control were done in a model of laser-induced choroidal neovascularization and intratumoral injections were performed in MBA-MD-231 xenografts in nude mice. VEGFR1_MOe13 elevated sFlt-1 mRNA expression and suppressed mbFlt-1 mRNA expression *in vitro* in multiple cellular backgrounds (p<0.001). VEGFR1_MOe13 also elevated sFlt/mbFlt-1 ratio *in vivo* after laser choroidal injury 5.5 fold (p<0.001) and suppressed laser-induced CNV by 50% (p = 0.0179). This latter effect was reversed by RNAi of sFlt-1, confirming specificity of morpholino activity through up-regulation of sFlt-1. In the xenograft model, VEGFR1_MOe13 regressed tumor volume by 88.9%, increased sFlt-1 mRNA expression, and reduced vascular density by 50% relative to control morpholino treatment (p<0.05).

**Conclusions:**

Morpholino oligomers targeting the VEGFR1 mRNA exon/intron 13 junction promote production of soluble FLT-1 over membrane bound FLT-1, resulting in suppression of lesional volume in laser induced CNV and breast adenocarcinoma. Thus, morpholino manipulation of alternative splicing offers translational potential for therapy of angiogenic disorders.

## Introduction

Angiogenesis, though a fundamental physiologic process, is a key pathogenetic feature of numerous disease states. At present, therapeutic strategies have limited potential largely due to the fact that the underlying mechanisms of angiogenesis are incompletely understood. Work to elucidate the full complement of mediators and mechanisms important for angiogeneis, and further apply this knowledge in such a way to alter disease progression continues to be the foremost goal within the field.

Identification of VEGF as a critical mediator of vessel growth has been an important step to understanding the human condition in terms of the underlying molecular events. VEGF has been shown to be necessary and sufficient for ocular neovascularization [1]–[4]. In fact, transgenic mice which over-express human VEGF show widespread ocular neovascularization [5]–[6]. Additionally, pioneering work by Judah Folkman in the early 1970's demonstrated that solid tumor growth required VEGF expression [7]. However, it is not fully known what specific molecular mediators regulate VEGF expression. Soluble Flt-1, first described by Kendall and Thomas in 1993, is an alternatively spliced form of the *Flt-1* gene, also referred to as VEGF-receptor 1 [8]. This alternative splicing event occurs within intron 13 such that sFlt-1 contains the ligand-binding domain, but lacks the membrane-spanning and intracellular kinase domains of the full-length membrane bound Flt-1 (mbFlt-1) protein [9]. As suggested by its structure, sFlt-1 is a potent endogenous inhibitor of VEGF A-induced angiogenesis [9]. While the full complement of sFlt-1 expression and function has not been described, it has been shown to be both necessary and sufficient for maintenance of the avascular cornea [10]. In addition, recent work has demonstrated a role for modulation of sFlt-1 in the development and treatment of a form of pathologic ocular neovascularization termed choroidal neovasculization (CNV), via modulation of VEGF ([11]–[12], unpublished data). Choroidal neovascularization is characterized by choroidal capillary growth through Bruch's membrane beneath the retinal pigmented epithelial (RPE) cell layer. This vascular pathology is most classically seen in exudative or “wet” age-related macular degeneration, the leading cause of vision loss in the western world [13]–[14]. Furthermore, sFLT-1 has also been shown to reduce VEGF expression and tumor vascularity in breast adenocarcinoma xenografts [15]. Thus, sFlt-1 represents a potential therapeutic target to reduce aberrant blood vessel growth over a spectrum of disease.

Current therapies targeting both CNV and tumor vasculature are focused on inhibiting the new vessel growth, and include such modalities as photocoagulation, photodynamic therapy, anti-VEGF intraocular injections, as well as systemic administration of anti-VEGF monoclonal antibody. These approaches have shown promise; however, they induce retinal damage, require repeated intraocular administration, or have recently been contraindicated for use in the case of systemic anti-VEGF therapy for breast adencarcinoma [4], [13], [16]–[17]. Additionally though certainly progress has been made, incomplete efficacy and recurrence is commonly seen with all modalities. Development of novel therapeutic techniques to either augment or circumvent our current treatments is necessary to improve both efficacy and the risk profile.

In this work, we describe the use of morpholino oligomers promoting the expression of soluble Flt-1 as a means to determine its potential for therapeutic use in disorders characterized by aberrant neovascularization. This technology has human precedent in Duchene Muscular Dystrophy, where morpholino technology targeting the dystrophin gene has shown efficacy in splice site alteration and disease modification in both animal and human trials [18]–[20]. Our work demonstrates the use of morpholino oligomers alters the splicing of VEGFR1 such that production of soluble Flt-1 is favored both *in vitro* and *in vivo*. Furthermore, we demonstrate the efficacy of increased sFlt-1 expression in treatment of aberrant blood vessel growth in ocular and cancer disease models. Thus, morpholino technology holds promise for use in the context of human disease.

## Results

### VEGFR1_MOe13 promotes a shift from membrane VEGFR1 to soluble VEGFR1 *in vitro*

**Figure 1:**
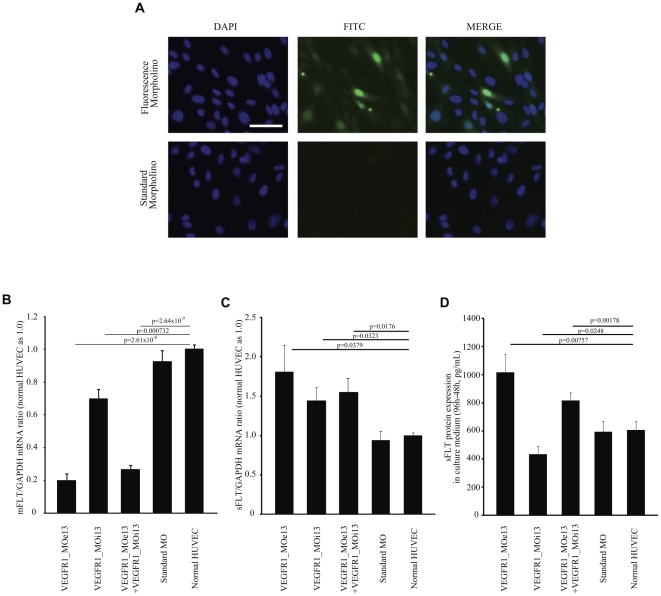
VEGFR1_MOe13 localizes to the nucleus and increases sFLT-1 expression in human endothelial vein cells (HUVEC). (a) Fluorescently tagged VEGFR1_MOe13 (F-MO) or standard morpholino (std-MO) were electroporated into HUVECs. After 48 hours fluorescence was assessed using light microscopy. Colocalization with DAPI staining represents nuclear localization of morpholino constructs. HUVECs were electroporated with VEGFR1_MOe13, VEGFR1_MOi13, a combination of VEGFR1_MOe13 and VEGFR1_MOi13, Standard_MO. All morpholino sequences were designed to target the human VEGFR1 transcript. (b) mbFLT-1 mRNA (n = 6) or (c) sFLT-1 mRNA expression (n = 6) were assessed using real time PCR. Values were normalized to GAPDH mRNA and normal HUVEC was used as 1.0. (d) sFLT protein expression in culture medium was determined by ELISA (n = 3). Data shows sFLT protein at 96 h – 48 h. Error bar is S.E.M. Each p-value was calculated by two-tail student's t-test against normal HUVEC.

In order to demonstrate the utility of morpholino constructs for modulating sFLT-1 expression, morpholino oligomers were designed targeting the FLT-1 mRNA exon13-intron13 junction (VEGFR1_MOe13) or intron13_exon14 junction (VEGFR1_MOi13). The canonical Flt-1 gene consists of thirty exons in human and mouse. Full-length mRNA from all exons produces mbFLT-1. By contrast, sFLT-1 utilizes a polyadenylation site within intron13. Therefore, interaction between the morpholino constructs and VegfR1 pre-mRNA is predicted to influence the alternative splicing event such that production of soluble FLT-1 is favored. To directly measure the relationship between membrane bound and soluble FLT-1 in the presence of VEGFR1_MOe13 and VEGFR1_MOi13, human umbilical vein endothelial cells (HUVEC) were electroporated with targeting or standard morpholino oligomers designed against human VEGFR1. Using this technique MO constructs were found to sufficiently access the nuclear compartment (Fig. 1a). Forty-eight hours after electroporation, total RNA was harvested and soluble VEGFR1 and membrane VEGFR1 mRNA expression was assessed using real-time PCR. We found that VEGFR1_MOe13, VEGFR1_MOi13, and a combination of a combination of one-half dose of both VEGFR1_MOe13 and VEGFR1_MOi13 significantly decreased membrane VEGFR1 mRNA and increased soluble VEGFR1 mRNA (Fig. 1b–c; Figure S1). To determine if the VEGFR1 morpholino constructs also affected soluble FLT-1 expression at the protein level, soluble VEGFR1 protein in HUVEC culture medium was measured by ELISA in the presence or absence of the VEGFR1 morpholino constructs or control mopholino. VEGFR1_MOe13 increased soluble VEGFR1 protein production as compared with standard morpholino or control PBS conditions (Fig. 1d). In contrast, VEGFR1_MOi13 were less effective, whereas combined delivery of both VEGFR1_MOi13 and VEGFR1_MOe13 showed intermediate response, possibly due to competitive interference or reduced efficacy of one morpholino as compared with the other. Thus, morpholino targeting of the FLT-1 mRNA exon13-intron13 junction using VEGFR1_MOe13 is most efficacious for increasing sFLT-1 and decreasing mbFLT-1 expression *in vitro*.

**Figure 2:**
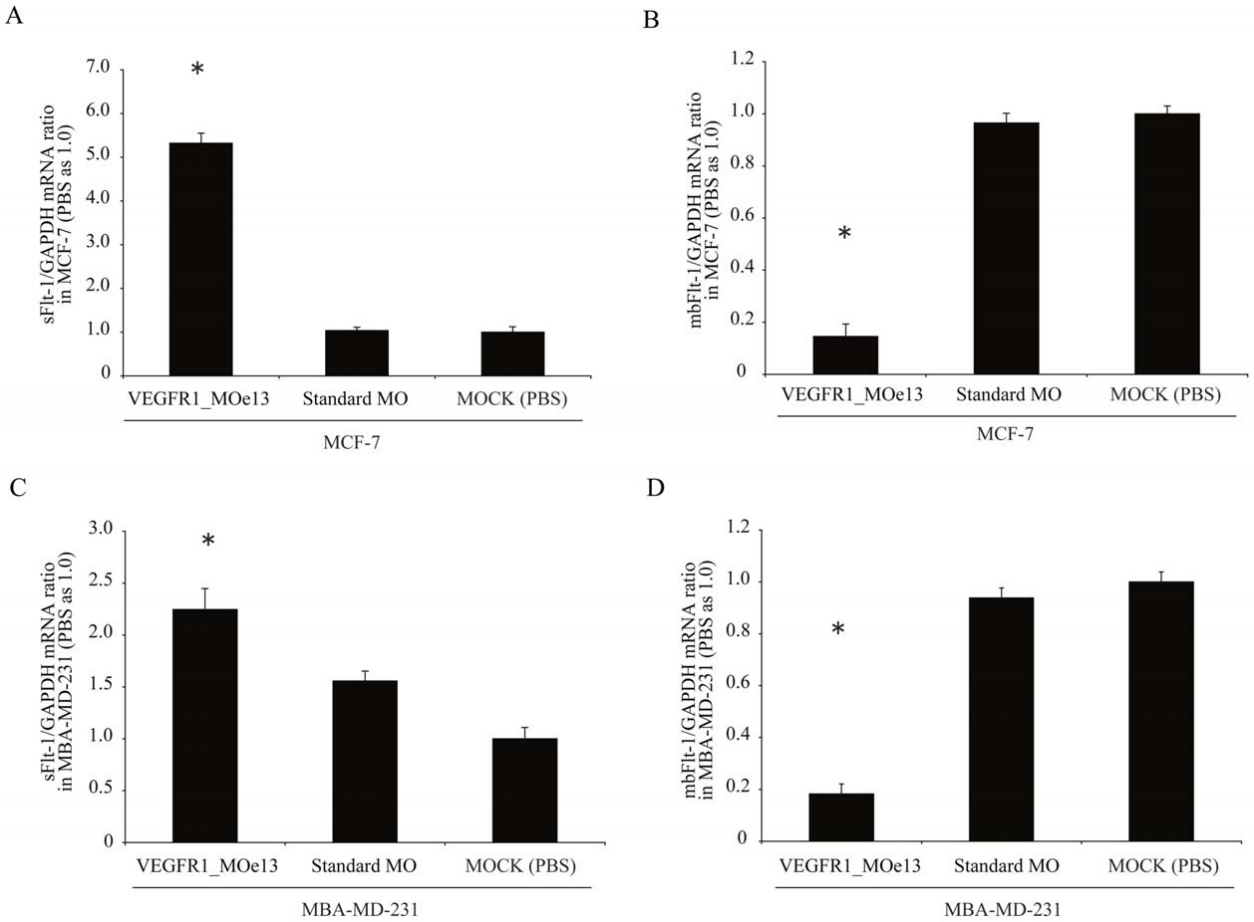
VEGFR1_MOe13 increases sFLT-1 and decreases mbFLT-1 mRNA in MCF-7 and MBA-MD-231 breast adenocarcinoma cell lines. MCF7 or MBA-MD-231 human breast adenocarcinoma cells were electroporated with VEGFR1_MOe13 and (a; c) sFLT-1 and (b; d) mbFLT-1 mRNA levels assessed at 72 hours using real time PCR (n = 3). Data were normalized to GAPDH mRNA levels and normal MCF7 or MBA-MD-231 cells were used a 1.0. *p<0.01.

To ensure that this effect was not cell line specific, we sought to validate our findings in other cellular backgrounds. Although soluble FLT-1 is predominantly a product of endothelial cells per its role in angiogenesis, it is also expressed by a number of other cell types including tumor cells [15], [21]–[24]. Thus, we sought to determine whether expression of the VEGFR1_MOe13 in MCF7 and MBA-MD-231 breast adenocarcinoma cells would increase soluble FLT-1 levels. As demonstrated in figure 2, electroporation of VEGFR1_MOe13 directed against the human FLT-1 transcript increases soluble FLT-1 RNA and decreases membrane FLT-1 RNA in both adenocarcinoma lines (Fig. 2a–d).

### VEGFR1_MOe13 increases sFlt/mFlt mRNA ratio in the mouse retina and suppresses laser-induced choroidal neovascularization volume *in vivo*

**Figure 3:**
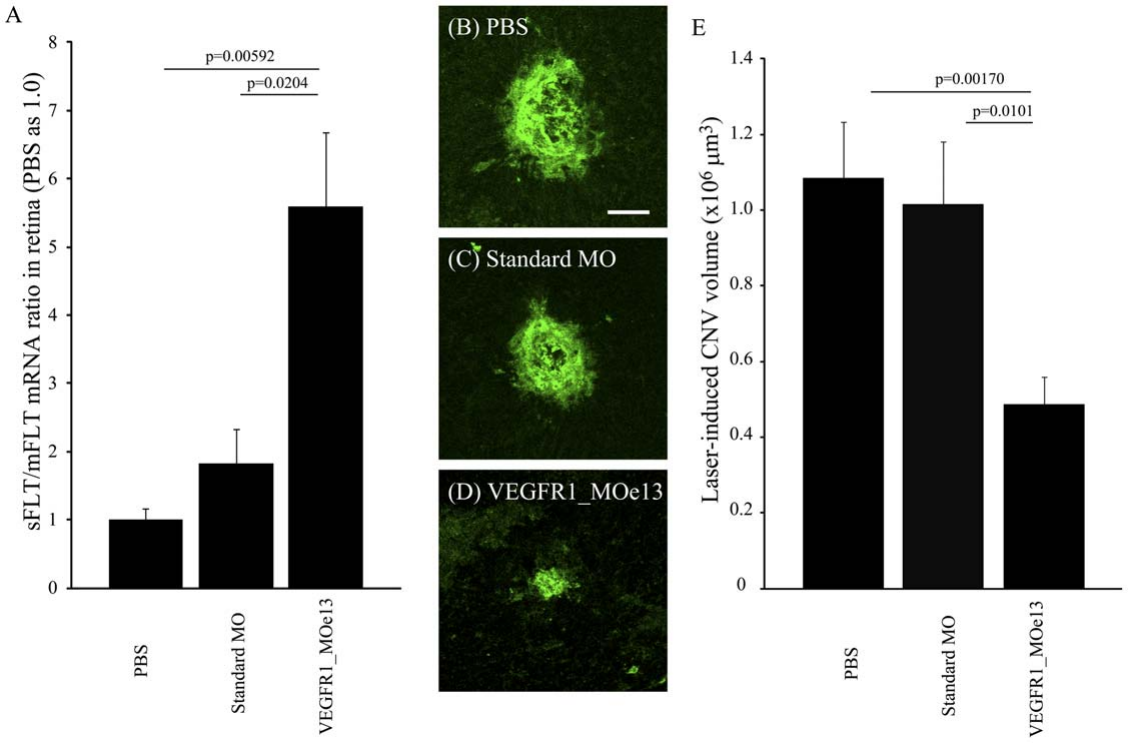
VEGFR1_MOe13 inhibits laser-induced CNV in vivo. a. sFlt/mFlt mRNA ratio in the retina treated with PBS, Standard_MO and VEGFR1_MOe13 (n = 4). Representative images of laser CNV injected with b PBS, c Standard_MO and d VEGFR1_MOe13 designed to target the murine VEGFR1 transcript. e The averages of laser CNV volumes (n = 11–14). Error bar is S.E.M. p-values were calculated by two-tail student's t test.

In order to directly test the efficacy of the VEGFR1_MOe13 for both *in vivo* activity as well as predicted effect on the process of angiogenesis we adopted the well established murine model of laser-induced choroidal neovascularization which induces significant CNV lesions 1 week after laser injury [25]–[26]. We hypothesized that expression of VEGFR1_MOe13 *in vivo* would both increase soluble Flt-1 levels and lead to suppression of laser-induced CNV. To first evaluate the effectiveness of VEGFR1_MOe13 to modulate sFlt-1 levels *in vivo*, we examined the sFlt/mFlt mRNA ratio in the mouse retina 24 hours after injection with PBS, vivo-standard_MO, or vivo-VEGFR1_MOe13 designed to target murine sFlt-1 (the “vivo” denotes modification allowing MO construct to enter cells in vivo as demonstrated [27]). We found that intra-vitreol injection of vivo-VEGFR1_MOe13 leads to a significant increase of sFlt/mFlt mRNA ratio as compared with PBS or vivo-standard_MO injection (Fig. 3a). Thus, VEGFR1_MOe13 expression is sufficient to increase soluble Flt-1 expression *in vivo*.

To determine whether VEGFR1_MOe13 expression *in vivo* could suppress development of CNV in the setting of laser insult, vivo-VEGFR1_MOe13, vivo-standard_MO or PBS were injected intra-vitreously on day 1 and day 4 after laser photocoagulation. One week after laser photocoagulation, eyes were enucleated and the degree of CNV volume was measured by confocal microscope after isolectin GS-IB_4_ vasculature staining. CNV volumes were quantified using confocal microscopy. Murine eyes treated with intra-vitreal vivo-VEGFR1_MOe13 displayed a statistically significant decrease in CNV volume as compared with eyes treated with either vivo-standard morpholino or PBS controls (Fig. 3b–d). Thus, intra-vitreol injection of vivo-VEGFR1_MOe13 leads to increased levels of sFlt-1 and suppression of laser-induced CNV.

### Short hairpin RNA-mediated sFlt-1 knock-down “rescues” the CNV phenotype *in vivo*

**Figure 4:**
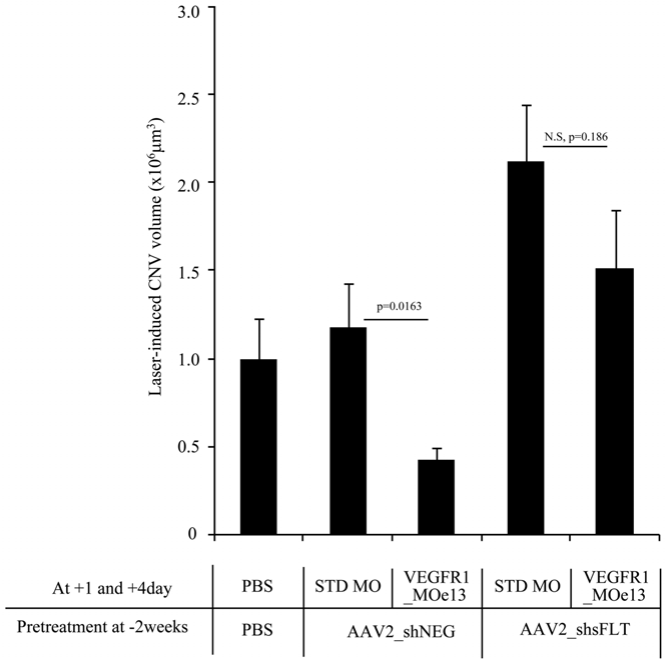
RNAi targeting sFlt-1 rescues the neovascular phenotype response to laser injury. Murine eyes were treated with PBS, AAV2_shNEG or AAV2_shsFLT. After 2 weeks, laser photocoagulation was performed. On 1 day and 4 day after photocoagulation, PBS Standard_MO or VEGFR1_MOe13 were injected. Error bar is S.E.M. N = 9–15.

In order to demonstrate that the measured effect of reduced CNV following intra-vitreol injection of VEGFR1_MOe13 was specific for an increase in sFlt-1 expression, we knocked-down sFlt-1 expression AAV2_shsFlt encoding short hairpin RNA(shRNA) targeting sFlt-1 mRNA (unpublished data; [10]). Intra-vitreol injections were performed using PBS, AAV2_shNEG (non specific shRNA) or AAV2_shsFlt (shRNA targeting sFlt-1) and laser photocoagulation performed 2 weeks later. In a consistent fashion with prior studies, on day 1 and 4 following photocoagulation PBS, vivo-standard_MO or vivo-VEGFR1_MOe13 constructs were injected into pretreated eyes. We hypothesized that if increased sFlt-1 was sufficient for suppression of the laser-induced CNV phenotype, co-expression of AAV2_shRNA_sFlt would reverse this effect. In agreement with this hypothesis, we observed that pre-treatment with AAV2_shsFlt results in reversal of VEGFR1_MOe13-mediated CNV suppression (Fig. 4). Thus, increased soluble Flt-1 expression is sufficient to at least partially mediate CNV suppression in the setting of laser-induced injury.

### Treatment of established MBA-MD-231 human breast adenocarcinoma xenograft tumors with VEGFR1_MOe13 results in tumor regression

**Figure 5:**
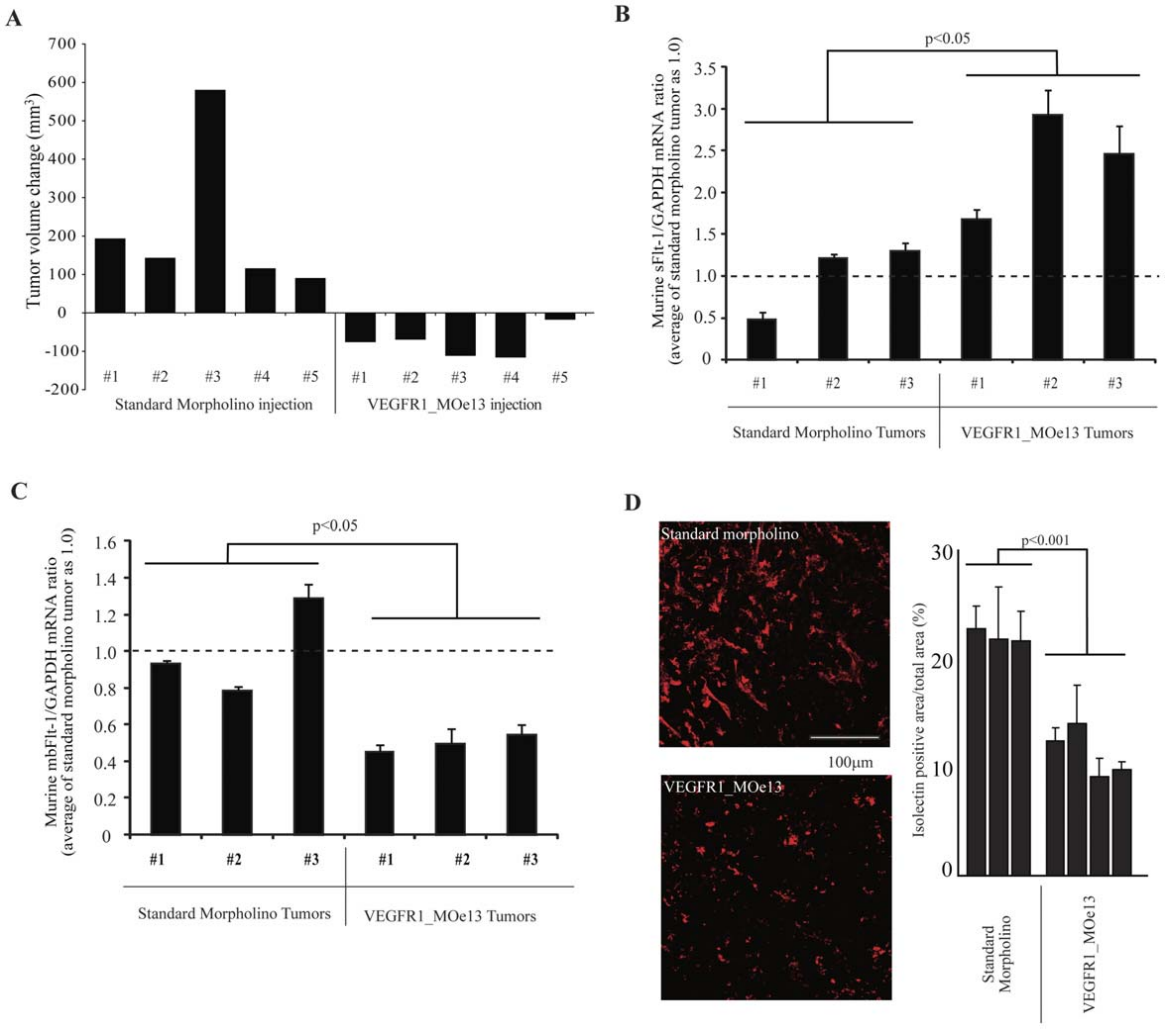
Intra-tumoral VEGFR1_MOe13 injection results in regression of established MBA-MD-231 human breast adenocarcinoma xenograft tumors and decreased tumor vascularity. a. MB-MDA-231 breast cancer cells were grown as xenografts in female nude mice for 2 weeks prior to beginning treatment with either a standard morpholino or VEGFR1_MOe13 designed to target the murine VEGFR1 transcript. Morpholino treated tumors demonstrate size regression after a 4 week treatment course as demonstrated by volume change analysis of 5 individual tumors within each treatment condition. p = 0.04 b–c. Following treatment course, RNA was extracted from xenograft tumors and soluble or membrane bound Flt1 levels were measured using real-time RT-PCR. Error bars indicate variation between individual PCR reactions per tumor sample. d. Following the treatment course, VEGFR1_MOe13 or standard morpholino xenograft tumors were sectioned and stained with isolectin as a measure of vascularity. Individual bars represent sections analyzed thoughout each tumor sample.

In order to demonstrate that anti-angiogenic activity of the VEGFR1_MOe13 construct is not limited to the ocular compartment, we sought to measure its efficacy in the setting of malignancy-associated neovascularization. Tumor vasculature is a rapidly emerging therapeutic target. As such, this model represents an attractive context in which to apply technologies designed to inhibit neovascularization. Within the context of malignancy, breast adenocarcinoma is known to demonstrate marked dependence on VEGF signaling for sustained neovascularization and growth [28]–[29]. In fact, VEGF inhibition has been shown to reduce tumor growth in both the experimental as well as the clinical setting [17], [30]. We hypothesized that treatment of MBA-MD-231 human breast adenocarcinoma xenograft tumors with vivo-VEGFR1_MOe13 would result in increased levels of soluble Flt-1 and a subsequent decrease in neovascularization and tumor regression. To directly test this hypothesis, female nude mice were inoculated as described. Xenografts were permitted to grow for 14 days. Tumors were then directly injected with either murine vivo-VEGFR1_MOe13 or vivo-standard morpholino. Injections and tumor volume assessments were performed bi-weekly for a duration of 4 weeks. We found that treatment of xenograft tumors with vivo-VEGFR1_MOe13 led to tumor regression when compared with standard morpholino treatment (p = 0.04) (Fig. 5a). Furthermore, murine sFlt-1 mRNA transcript levels were increased and mbFlt-1 mRNA levels decreased in treatment tumors at the conclusion of the 4 week treatment period as compared with control tumors when assessed using real-time PCR (Fig. 5b–c). Finally, to determine whether vascular density was decreased in tumors treated with viv-VEGFR1_MOe13 injection, tumor vasculature was stained with GS-IB_4_ and vessel density quantified using fluorescence microscopy following the 4 week treatment period. Tumors treated with the vivo-VEGFR1_MOe13 construct demonstrated a statistically significant decrease in vascular density (Fig. 5d). Interestingly, these results were achieved using VEGFR1_MOe13 targeting the murine Flt-1 transcript, while treatment with a VEGFR1_MOe13 construct targeting human FLT-1 did not demonstrate tumor regression *in vivo* (Figure S2). This may indicate that host vasculature is important for tumor growth,, suggesting that human sFLT-1 produced by tumor cells does not effectively inhibit murine VEGF signaling. However, analysis of the human and murine morpholino sequences demonstrates significant overlap between the murine morpholino construct with the human VEGFR1 sequence. The same is not true for the human morpholino with respect to the murine VEGFR1 mRNA sequence. Therefore, this cross-reactivity could account for our findings as could overall decreased efficacy of the human morpholino construct. The latter is unclear given the fact that *in vitro* this sequence increases soluble FLT-1 very effectively. Further studies are needed to fully understand this.

## Discussion

Neovascularization is a common pathological process underpinning numerous disease states. Elucidation of key molecular mediators has allowed for the development of therapeutic strategies targeting the underlying molecular processes. Soluble Flt-1 first described in 1993, is an alternatively spliced form of the *Flt-1* gene [4]–[5]. The morpholino oligomer VEGFR1_MOe13 is designed to target the Flt-1 mRNA exon13-intron13; therefore, interaction between VEGFR1_MOe13 and VegfR1 pre-mRNA is predicted to influence the alternative splicing event such that production of soluble Flt-1 is promoted.

Herein we demonstrate that expression of VEGFR1_MOe13 both *in vitro* and *in vivo* results in elevated levels of soluble Flt-1 and decreased membrane bound Flt-1. Furthermore, these data show that VEGFR1_MOe13-mediated increase in soluble Flt-1 within the retina can prevent choroidal neovascularization following laser photocoagulation. Finally, our data indicate that this phenotype is attributable to increased sFlt-1 expression as co-treatment with sFlt-1-RNAi negates this effect. Thus, soluble Flt-1 expression is both necessary and sufficient for suppression of laser-induced CNV. Taken together these data indicate that modulation of soluble Flt-1 expression in the clinical setting has potential therapeutic value. This represents great promise when considering the over 7 million patients in the US alone with non-exudative age related macular degeneration (ARMD) currently “at risk” for development of CNV.

Angiogenesis in the form of aberrant neovascularization is fundamental to the pathophysiology of other disease states as well. Notably, new blood vessel formation is absolutely required for sustained solid tumor growth [7]. As noted, breast adenocarcinoma is dependent on sustained neovascularization both in animal and human studies [28]–[29]. As a “proof of principle” for broad applicability of morpholino-mediated Flt-1 modulation in the treatment of neovascular disease, we demonstrate that intra-tumoral injection of VEGFR1_MOe13 leads to increased intra-tumoral levels of sFlt-1 and decreased mbFlt-1.. Furthermore, breast adenocarcinoma xenografts expressing elevated levels of sFlt-1 demonstrate a blunted neovascular response and regress once established as compared with control tumors. Thus, modulation of soluble Flt-1 expression using morpholino technology represents a therapeutic tool with broad applicability across a spectrum of neovascular disease.

Taken together, these data indicate that morpholino expression is a viable tool for modulating the expression of FLT-1, i.e., the balance between membrane and soluble forms of this transcript. In our system, these data indicate that morpholino interference at the Flt-1 mRNA exon13-intron13 junction leads to the greatest amount of soluble FLT1 production. The VEGFR1_MOe13 construct is effective in two independent disease models, choroidal neovascularization and breast adenocarcinoma. In both, aberrant vasculature and disease burden are reduced. Given the highly divergent roles of FLT1 isoforms, morpholino oligos show promise in a variety of physiologic settings and disease states to act as an “exogenous switch” to modulate angiogenesis.

These data demonstrate the efficacy of morpholino technology to influence FLT-1 gene expression at the mRNA level both *in vitro* and *in vivo* setting. Recent application of splice-blocking morpholinos in the setting of DMD has demonstrated disease-modifying effects in both animal and human trials [18]–[20]. Therefore, these constructs hold promise as true “bench to bedside” tools. Within the setting of neovascularization, use of morpholino technology represents a shift from current therapies which are predominantly based on monoclonal antibody approaches. The evolving potential for morpholino technology to be targeted to areas of pathology following systemic administration may lend this technology to broader appeal and utility [31]. Additionally, combining morpholino-mediated sFLT-1 modulation with other known anti-angiogenic strategies is a promising area of investigation. For example, morpholino-based inhibition of other angiogenic factors such as FGF and PDGF remains an interesting question, as these factors have been shown to promote intra-ocular neovascularization and inhibition shown to reduce neovascular pathology [32]–[34]. Furthermore, synergy between these pathways has been shown to promote tumor vascularity and metastasis in murine studies [35]–[36]. Therefore, steric inhibition of splice sites required for proper expression of these factors may represent yet another role for morpholino technology in the treatment of neovascular disease. Thus morpholino technology currently represents an effective mechanism for treatment of neovascular pathology across a spectrum of disease with high translational potential and exciting future applications.

## Materials and Methods

### Constructs and Reagents

*In vivo* morpholino constructs were designed to target murine intron/exon 13 junction of the FLT-1 transcript denoted VEGFR1_MOe13 and VEGFR1_MOi13. Constructs were chemically modified such that they would easily diffuse to access the intracellular compartment [27]. For use in xenograft assays, VEGFR1_MOe13 morpholino constructs were suspended in sterile PBS and used at a concentration of 400 ng/dose. Each dose is equal to 50 ul total volume. A standard morpholino targeting the murine beta-globin subunit2 was used as a control and prepared at the same concentration. Morpholino sequences are as follows.

Human VEGFR1_MOe13 GTTGCAGTGCTCACCTCTGATTGTA

Human i13 GCTTCCTGATCTAGTGAAGAAAGAA

Mouse VEGFR1_MOe13 CTTTTTGCCGCAGTGCTCACCTCTA

STD MO CCTCTTACCTCAGTTACAATTTATA

AAV.shRNA.sFlt-1 was developed as previously described [11]. 2.5×10∧9 GC of AAV.shRNA.sFlt-1 were injected per experimental condition.

### Cell culture

Primary human umbilical vein endothelial cells (Lonza, Walkersville, MD, USA) were cultured in EBM with EGM SingleQuot Kit supplements and growth factors according to the manufacturer's instructions (Lonza, Walkersville, MD, USA). To prevent loss of endothelial cell properties, cell cultures were limited to passage number 4 to 7. MBA-MD-231 human breast adenocarcinoma cells were obtained from the laboratory of Dr. Bryan Welm MD at the Huntsman Cancer Institute, SLC, Utah and maintained in RPMI culture medium containing 10% FBS. MCF7 human breast adenocarcinoma cells were obtained for the laboratory of Dr. Alana Welm PhD at the Huntsman Cancer Institute, SLC, Utah and maintained per ATCC medium recommendations.

### Morpholino delivery to cultured cells and total RNA extraction

10 ng of morpholinos were delivered to the nucleus by nucleofection (Amaxa, Gaithersburg, MD, USA) using a Basic Nucleofector Kit for Primary Mammalian Endothelial Cells (Amaxa, Gaithersburg, MD, USA), program A-034 for HUVEC. For one nucleofection, 1×106 cells were used and plated on a 6-well plate. After 2 days of culture, cells were trypsinized and total RNA was extracted using a RNeasy mini kit (Qiagen, Valencia, CA, USA)]. RNA concentrations were determined by 260 nm absorption measured with a spectrophotometer.

### Complementary DNA synthesis and quantification with real-time PCR

Complementary DNA (cDNA) were synthesized from approximately 400 ng total RNA using an Omniscript RT kit (Qiagen, Valencia, CA, USA) and Oligo-dT primer (dT2) according to the manufacturer's instructions. Real-time PCR was performed using a QuantiTect SYBR Green PCR Kit (Qiagen, Valencia, CA, USA) and an aliquot 1∶l of cDNA solution. The primer sequences used for human sequence real-time PCR were:

VEGFR1_F1: 5′-CTGCAAGATTCAGGCACCTA-3′

VEGFR1_R1: 5′- CCTTTTTGTTGCAGTGCTCA-3′

VEGFR1_F2: 5′-AACCAGAAGGGCTCTGTGGAAAGT-3′

VEGFR1_R2: 5′-CAAACTCCCACTTGCTGGCATCAT-3′

The combination of VEGFR1_F1 and R1 was designed to detect the soluble form of human VEGFR1. The combination of VEGFR1_F2 and R2 was designed to detect the membrane form of human VEGFR1 .

The primer sequences used for murine sequence real-time PCR were:

VEGFR1_F1: 5′ –AATGGCCACCACTCAAGATT

VEGFR1_R1: 5′ –TTGGAGATCCGAGAGAAAATG

VEGFR1 F2: 5′ –GATCAAGTTCCCCTGGATGA

VEGFR1_R2: 5′ –ATGCAGAGGCTTGAACGACT

Gapdh_F: 5′ –AACTTTGGCATTGTGGAAGGGCTC

Gapdh_R: 5′ -ACCAGTGGATGCAGGGATGATGTT

The combination of VEGFR1_F1 and R1 was designed to detect the soluble form of murine VEGFR1. The combination of VEGFR1_F2 and R2 was designed to detect the membrane form of murine VEGFR1 .

Real-time PCR conditions: 50°C for 2 minutes, 95°C for 10 minutes, followed by 40 cycles of 94°C for 15 seconds, 55°C for 30 seconds, and 72°C for 30 seconds.

### Laser induced choroidal neovascularization

Laser photocoagulation (532 nm, 150 mV, 100 ms, 100 µm; NIDEK MC-4000) was performed on both eyes (2 to 5 spots per eye) as described [24]–[25]. After enucleating the eyes, sclera/choroid/RPE complex were fixed in 4% paraformaldehyde for 2 hours at 4°C. After blocking in 5% FBS/PBS with 0.02% tritonX-100 and 2 mM MgCl2, samples were stained with 5 µg/ml Alexa488 and Alexa546 conjugated isolectin GS-IB_4_ (Invitrogen Corporation, Carlsbad, USA) overnight. After washing the samples were flat mounted on glass slides. CNV volume was measured by scanning laser confocal microscopy (Olympus America Inc., Center Valley, USA). These animal studies were performed under IACUC protocol approval number 09-03005 approved by the Committee on the Ethics of Animal Experiments at the University of Utah. All interventions were performed either under sodium pentobarbital anesthesia or after animals were humanely euthanized.

### Xenograft analysis

3×10^6^ MBA-MD-231 human breast adenocarcinoma cells were injected subcutaneously into the flanks of female nude mice. Following a standardized 2 week inoculation period, xenograft tumors were injected biweekly for a total duration of 4 weeks with VEGFR1_MOe13 or standard morpholino control. These constructs were prepared as detailed above. Tumor growth was assessed using digital calipers with bi-weekly measurements correlating with injection period. (Volume mm^3^ = width^2^×length/2. These animal studies were performed under IACUC protocol approval number 09-03005 approved by the Committee on the Ethics of Animal Experiments at the University of Utah. All interventions were performed either under sodium pentobarbital anesthesia or after animals were humanely euthanized.

### Immunohistochemical anlaysis

Tumors were fixed in 4% paraformaldehyde for 2 h at 4 C, cryoprotected in 15% sucrose and 30% sucrose, and then embedded in OCT (Tissue-Tek, USA). Sections (12 um) were cut and were incubated with isolectin (Griffonia simplicifolia, Alexa Fluo568 conjugate 1∶1000, Invitrogen Corporation, Carlsbad, CA) overnight. Immunohistochemistry results were examined using scanning laser confocal microscopy (Olympus, FLUOVIEW, FV1000, 20×). These images were scored by Image-J morphometry system using biometry scoring (Wayne Rasband).

After electroporation of F-MO(10 ul of 1 mM) or STD MO into HUVECs, the cells were plated to 8 well glass slide (nunc, Rochester, NY) coated with collagen. 24 hours later, the cells were fixed with 4% parafolmaldehyde/PBS, stained with DAPI, and observed with the Zeiss Axiovert 200 inverted fluorescence microscope.

### Statistical analysis

Data are presented as mean +/−SEM. Statistical analysis was performed using student T test. A p-value 0.05 was considered significant.

## Supporting Information

Figure S1 The Soluble/Membrane FLT-1 ratio increases following electroporation of VEGFR1_MOe13 into HUVEC. HUVECs were electroporated with VEGFR1_MOe13, VEGFR1_MOi13, a combination of VEGFR1_MOe13 and VEGFR1_MOi13, Standard_MO. All morpholino sequences were designed to target the human VEGFR1 transcript. mbFLT-1 mRNA and sFLT-1 mRNA expression were assessed using real time PCR. Values were normalized to GAPDH mRNA and normal HUVEC was used as 1.0.
(TIF)

Figure S2 Intra-tumoral injection of VEGFR1_MOe13 targeting human FLT-1 does not induce xenograft tumor regression. MB-MDA-231 breast cancer cells were grown as xenografts in female nude mice for 2 weeks prior to beginning intra-tumoral injection treatment with either a standard morpholino or VEGFR1_MOe13 morpholino targeting the human FLT-1 transcript. Change in tumor volume was assessed following a 4 week treatment course. p = 0.3
(TIF)
