# Supplementary figures and images for: Morpholino-Mediated Increase in Soluble Flt-1 Expression Results in Decreased Ocular and Tumor Neovascularization
Source: PLoS One. 2012 Mar 15;7(3):e33576. doi: 10.1371/journal.pone.0033576 (PMC3305322; doi:10.1371/journal.pone.0033576)

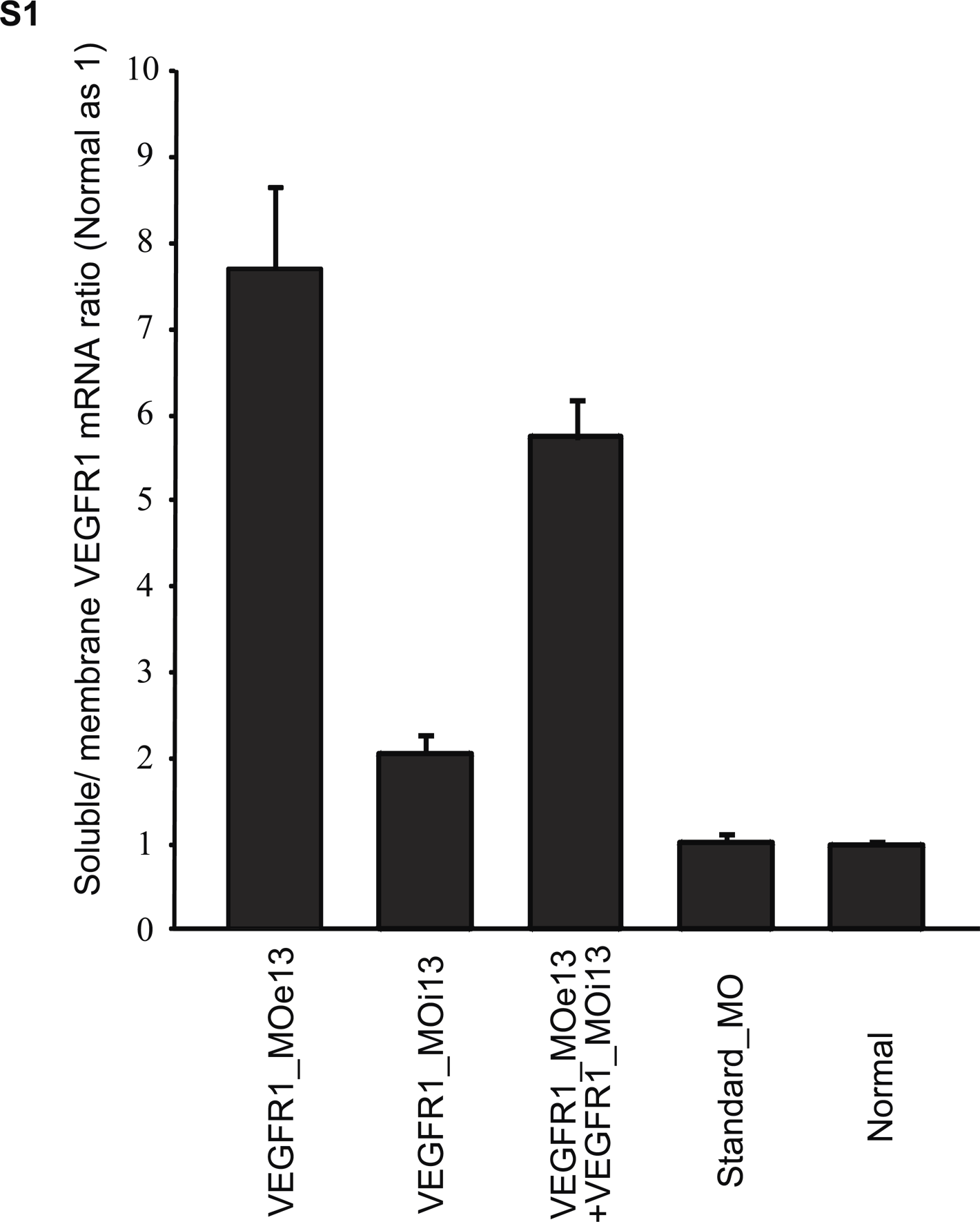

Supplement: Figure S1 — The Soluble/Membrane FLT-1 ratio increases following electroporation of VEGFR1_MOe13 into HUVEC. HUVECs were electroporated with VEGFR1_MOe13, VEGFR1_MOi13, a combination of VEGFR1_MOe13 and VEGFR1_MOi13, Standard_MO. All morpholino sequences were designed to target the human VEGFR1 transcript. mbFLT-1 mRNA and sFLT-1 mRNA expression were assessed using real time PCR. Values were normalized to GAPDH mRNA and normal HUVEC was used as 1.0. (TIF) [file pone.0033576.s001.tif]

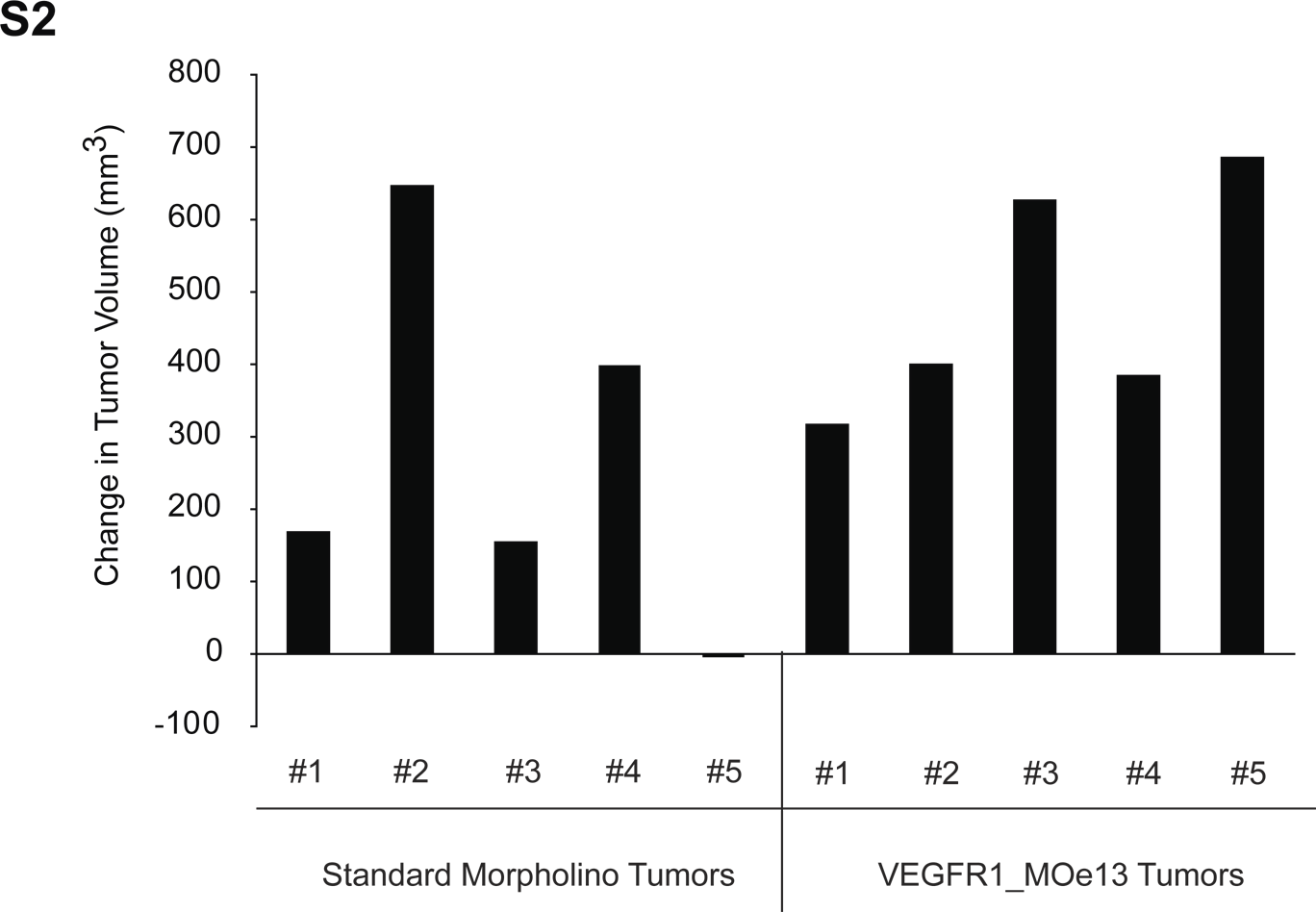

Supplement: Figure S2 — Intra-tumoral injection of VEGFR1_MOe13 targeting human FLT-1 does not induce xenograft tumor regression. MB-MDA-231 breast cancer cells were grown as xenografts in female nude mice for 2 weeks prior to beginning intra-tumoral injection treatment with either a standard morpholino or VEGFR1_MOe13 morpholino targeting the human FLT-1 transcript. Change in tumor volume was assessed following a 4 week treatment course. p = 0.3 (TIF) [file pone.0033576.s002.tif]
